# Dietary availability patterns of the brazilian macro-regions

**DOI:** 10.1186/1475-2891-10-79

**Published:** 2011-07-28

**Authors:** Sileia Nascimento, Flávia S Barbosa, Rosely Sichieri, Rosangela A Pereira

**Affiliations:** 1 Department of Social and Applied Nutrition, Institute of Nutrition Josué de Castro, Federal University of Rio de Janeiro, (Av. Carlos Chagas, 373, Edifício do Centro de Ciências da Saúde (CCS) - Bloco J - 2° andar - sala 10 - Cidade Universitária - Ilha do Fundão), Rio de Janeiro, (21941-902), Brazil; 2 Department of Epidemiology, Institute of Social Medicine, State Universityof Rio de Janeiro, (Rua S. Francisco Xavier, 524, 7o andar, Bloco E), Rio de Janeiro, (20550-012), Brazil

**Keywords:** dietary availability patterns, factor analysis, food availability

## Abstract

**Introduction:**

Epidemiological studies have raised concerns about the role of dietary patterns on the risk of chronic diseases and also in the formulation of better informed nutrition policies.

**Objective:**

The development of a dietary availability patterns according to geographic regions in Brazil.

**Methodology:**

The 2002-2003 Brazilian Household Budget Survey was conducted in 48,470 households. Dietary availability patterns were identified by Principal Component Analysis using as a unit of analysis the survey's Primary Sampling Units (PSUs) and purchased amounts for 21 food groups. Each of the extracted dietary availability patterns was regressed on socioeconomics categories.

**Results:**

There were no differences in dietary availability patterns between urban and rural areas. In all regions, a rice and beans pattern was identified. This pattern explained 15% to 28% of the variance dependent on the region of the country. In South, Southeast and Midwest regions, a mixed pattern including at least 10 food groups explaining 8% to 16% of the variance. In the North region (Amazon forest included) the first pattern was based on fish and nuts and then it was designed as regional pattern. In multiple linear regression the rice and beans pattern was associated with the presence of adolescents in the households, except for North region, whereas the presence of adolescents was associated with the Regional pattern. A mixed patterns were associated with a higher income and education (p < 0.05), except in the South region.

**Conclusion:**

The rice and beans and regional dietary availability patterns, both considered healthy eating patterns are still important in the country. Brazil has taken many actions to improve nutrition as part of their public health policies, the data of the Household Budget Survey could help to recognize the different food choices in the large regions of the country.

## Introduction

Identification of dietary patterns has been widely used to classify individuals and communities according to healthy and unhealthy diets, overcoming many restricted characteristic of traditional analyses focused on nutrient intake [1].

Few studies in Brazil have identified dietary patterns, and all of them were limited to local populations or restricted groups [2-4]. The identification of dietary patterns and respective associated factors based on representative data of the whole country allows the establishment of guidelines and policies to promote better and healthier food intake.

Obtained data on food intake in population-based studies is neither an easy nor an expensive task. Therefore, Household Budget Surveys (HBS) have been used as an alternative method for estimating dietary changes in the population. The HBS collect food availability data at the household level and stand a position between the food balance sheets and the specifically designed individual-based food consumption surveys. HBSs are country-representative surveys, conducted at regular time intervals by the National Statistical Office in Brazil and other countries [5]. In addition to estimating the index price, HBS in Brazil measure amounts of food acquisition, which is regularly updated using international standardized methodology [6]. HBS allow to estimate dietary availability patterns in households and then create the opportunity to test associations with the demographic and socioeconomic factors [5].

Among a few studies that have investigated the dietary availability pattern by analyzing data from the HBS, Naska et al. [7] applied to the Principal Component Analysis to examine the dietary availability patterns in ten European countries and observed two main patterns, the first dietary pattern availability was remarkably similar in all countries with positive loadings for a wide range of foods, from fruits, vegetables and cereals to meat, fish and dairy products, with rarely negative loading and it was interpreted as a mixed pattern, that indicates households with a wide variety of food purchasing. This pattern was more common among households whose head was retired and elderly people. The second pattern had positive loadings for beverages (alcoholic and nonalcoholic) and foods that could be consumed without elaborate preparation and negative loadings for plant foods and some requiring laborious kitchen work. This pattern was more common among households located in urban or semi urban areas and also among Scandinavian adults living alone.

The HBS study in South America was conducted in Bolivia to monitor food availability among 19,483 households, and results showed that the Bolivian diet is characterized by higher availability of foods of plant origin [8].

The objective of the present study was to describe the dietary availability patterns in the large Brazilian geographic regions based on HBS and examine the socioeconomic determinants that could be associated with these patterns in each region.

## Methods

### Study design

The present study was based on the nationwide 2002-2003 Brazilian HBS [9]. The study adopted a two-stage cluster sample design. The geographic sectors of the 2000 Brazilian Demographic Census were the primary sampling units (PSU). Sampling was stratified by the five regions of the country and by the socioeconomic level of PSU. Households were selected by simple random sampling without replacement within the selected PSUs. The number of households selected by PSU was of 28 households for the urban area PSU and of 30 to 34 for the rural area PSU. In order to capture seasonal variability, sample collection was distributed evenly throughout the year. The 2002/2003 HBS collected information from 4,000 PSUs, totalizing 48,470 households. In the present study, the PSUs with less than four households interviewed were excluded. A total of 3,974 PSUs were analyzed, totaling 48,315 households.

The data allowed assessing the dietary availability pattern of overall country, all states, and the large regions of the country. Information on the socio-demographic characteristics of the household members such as age, education of the head of the family, and income of the household was also collected [9].

### Data on food availability

The basic information of HBS is the purchases of food and beverages for household consumption during a period of seven followed days recorded daily by the participants and reviewed by trained interviewers. The records include a detailed description of the product, the amount purchased and the paid value.

Since HBS are not designed to primarily serve Nutrition purposes, the food data vary from very detailed records to more aggregated ones. Therefore, it became necessary to add food items to the lowest level of information (i.e. fishes classified under 900 different codes were added into three groups: freshwater fish, saltwater fish and unspecified).

A total of 5,442 food products were reported and further categorized in 21 food groups, based on the Food Guide for the Brazilian Population [10]. Items that were purchased for other units of consumption were excluded from the analyses; these represented less than 0.5% of the total of items.

For purchases such as rice, beans, sugar and oils usually bought monthly the total amount could varied from zero to many kilograms. Thus for all food groups amount it was divided by the number of households composing each PSU. In order to render the data more symmetrical, obtained the amounts acquired of the food groups were log transformed and PSU instead of household were used in the analysis. Following the logarithmic transformation, the mean quantities in kilos or liters were evaluated by attributing a score of 0 to 4, where 0 indicated no acquisition and 1 to 4 referred to the quartiles of distributions of the mean amounts purchased.

All variables were related to the PSUs, such as percentage of PSUs with children, adolescents, or elderly people, PSUs' mean income, and percentages of PSUs with education of the household head, categorized according to the educational level attained (illiterate, elementary school, high school, and college or more).

### Identification of dietary availability patterns and statistical analysis

In order to verify whether the data being analyzed were appropriate for the factorial analysis, the test of Kaiser-Meyer-Olkin (KMO) was used [11,12]. The dietary availability patterns by region were derived by means of an exploratory factor analysis - Principal Component Analysis - using the *Proc Factor *procedure. For a better interpretation of the factors obtained, these were submitted to orthogonal rotation by the Varimax method. To identify the number of factors to be retained two commonly applied criteria were used: (1) the eigenvalues >1.0 criterion, and (2) the graphical Cattell's scree test [12].

The factor loading indicates the importance of food group in the definition of the pattern, absolute scoring coefficients greater than 0.14 were considered as important contributors to a pattern. The internal consistency of the items that compose each pattern was evaluated by a Cronbach's Alpha coefficient and values greater than 0.6 were considered acceptable.

The factor loadings were standardized using the *Proc Standard *command, which standardizes the variables to a mean equal to 0 and standard deviation equal to 1 in order to make the multiple linear regression models comparables. Linear regression models included the patterns identified as dependent variables. The independent variables were: the percents of elderly people, children, and adolescents in the PSUs, the PSU's average monthly total household income and the percents in the education categories regarding the head of household (illiterate, elementary school, high school, college or more) in the PSUs.

The average monthly household income per *PSU *(in minimum wages) was categorized as: from 1 to 2, from 3 to 5, and >5 minimum wages. The legal minimum wage per month established for the country at the time of the 2002/2003 HBS was R$200, which corresponded to US$74.

All analyses were conducted using the SAS statistical software package version 9.1.

All ethical related questions are in accordance with the Brazilian Resolution Number 196/96- on research involving human issues. For all the census and surveys conducted by the bureau of census there is a specific federal law (law number 5534 from November 14, 1968) which guarantees top secret information.

## Results

High KMO values (>0.8) in all region analyses indicated the appropriateness of the factor analysis.

Characteristics of each macro-region of the country are described in table 1. The North and Northeast regions showed as the greatest differences in socio-demographic characteristics. South, Southeast, and Midwest regions were more homogeneous with only a few PSUs with income below two times the minimum wage.

**Table 1:** Sample size, socioeconomic and family composition variables of the Brazilian geographical macro-regions

	Brazil	North	Northeast	Southeast	South	Midwest
Number of PSUs*	3,974	554	1,512	707	506	695
Number of households	48,315	6,868	18,591	8,620	6,080	8,156
PSUs in rural areas	582	114	243	92	49	84
PSUs in urban areas	3,392	440	1,269	615	457	611
Income minimum wage** - %						
1 - 2	3.3	1.4	7.5	0.6	0.4	0.4
3 - 5	38.6	43.0	55.5	20.1	15.6	34.0
> 5	58.3	55.6	37.0	79.3	84.0	65.6
Education of the head of the family (%)						
Illiterate	3.4	3.6	5.4	1.5	1.2	2.1
Elementary	53.4	53.0	47.5	58.2	61.9	56.2
High school	18.1	21.5	16.0	17.7	19.5	19.3
Undergraduate and graduate	5.0	3.5	3.6	7.7	5.9	5.7
Children per PSUs (%)	17.9	21.7	18.1	15.6	15.8	17.5
Adolescents per PSUs (%)	21.1	23.1	22.4	19.0	18.5	19.7
Elderly per PSUs (%)	5.7	3.9	6.1	6.4	6.4	5.0

* Primary sampling unit

**US$ 74.00 (Seventy-four dollars per month)

In all macro-regions, one rice and beans pattern containing rice, beans, flours (wheat, manioc, and corn meal), oils, and caffeinated beverages (coffee, tea, and yerba mate) was detected (Table 2).

**Table 2:** Loading values according to dietary patterns extracted by principal components analyses of the geographic regions of Brazil

	North			Northeast			Southeast		South		Midwest	
**Food Groups**	**Regional**	**Rice and Beans**	**Fruit/Greens/Sweets**	**Rice and Beans**	**High Energy**	**High Protein**	**Rice and Beans**	**Mixed**	**Rice and Beans**	**Mixed**	**Rice and Beans**	**Mixed**

Rice		0.69		0.53			0.70		0.70		0.79	
Beans		0.56		0.65			0.69		0.69		0.74	
Vegetables and leafy vegetables			0.57			0.62		0.55		0.55		0.62
Potatoes			0.69		0.55			0.53		0.53		0.50
Others tuberous roots			0.37		0.52			0.38		0.38		0.57
Flours (wheat, manioc and corn meal)	0.69			0.55			0.76		0.76		0.56	
Fruits			0.50			0.53		0.64		0.64		0.65
Nuts and coconut	0.52					0.20		0.14		0.14		0.14
Pastry	0.44				0.61		0.47		0.47		0.55	
Breads	0.38				0.56			0.49		0.49		0.44
Cakes & cookies	0.32				0.49			0.55		0.55		0.46
Meats	0.45					0.58	0.52		0.52			0.35
Poultry and eggs	0.53					0.33	0.46		0.46			0.37
Fish	0.66					0.28		0.29		0.29		0.25
Milk and dairy		0.46			0.19			0.46		0.46		0.32
Butter and Margarine	0.51				0.60			0.39		0.39		0.39
Sugar		0.59		0.77			0.69		0.69		0.75	
Sweetened beverages			0.54		0.41			0.55		0.55		0.49
Oils and fats		0.72		0.68			0.70		0.70		0.77	
Caffeinated beverages		0.50		0.63			0.58		0.58		0.56	
Products diets/light			0.40		0.30			0.24		0.24		0.31
Eigenvalues	2.90	2.88	2.26	2.99	2.69	1.77	3.76	2.86	4.0	3.0	3.86	2.91
%Variance explained	20.4	14.6	11.6	19.6	16.1	8.0	22.4	15.2	27.1	12.3	25.2	12.3
Cronbach Coefficient Alpha	0.76	0.76	0.68	0.80	0.73	0.60	0.86	0.76	0.86	0.75	0.85	0.78

Kaiser's Measure of Sampling Adequacy	0.83			0.84			0.87		0.89		0.88	

National Household Budget Survey 2002/2003

Values < 0.15 were excluded for simplicity

In the North region the first pattern, which explained 20% of the variance, was characterized by the acquisition of foods characteristic of that region, such as fish and coconut named as regional pattern. The third pattern designated as fruits/greens/sweet pattern, explained 11.6% of the variance, included vegetables and leafy vegetables; potatoes; others tuberous roots; fruits; sweetened beverages and products diets/light (Table 2).

In the Northeast, the rice and beans pattern was the one that explained most of the variance (19.6%). A second pattern explained 16% of the variance and was characterized by the acquisition of potatoes, other tuberous roots, pasta, breads, cakes and cookies, milk and dairy, butter and margarine, and sweetened beverages (soft drinks, juices, and others), this pattern was designated as high energy pattern. The third pattern explained 8% of the variance and was interpreted as indicating PSUs with acquisition of the group foods as vegetables and leafy vegetables; fruits; nuts and coconuts; meats; poultry and eggs; fish. This pattern was denominated as the high protein pattern (Table 2).

The high energy pattern, in the Northeast region, was associated to elementary and high school education levels. For PSUs in which the household heads had an elevated mean of education in years, a greater adherence to the mixed patterns characterized by acquisition of fruits, vegetables and leafy vegetables, sugar, and diet and/or light products was detected, The same relationship with mixed pattern was observed for mean sector's income (Table 3).

**Table 3:** Multiple regression coefficients β (and 95% CIs) and R^2^, associated with the standards of the Brazilian regions according to the proportion of predictor variables within each PSU:

	North		
	**Regional** **β (CI 95%)**	**Rice and beans** **β (CI 95%)**	**Fruit/Greens/Sweets** **β (CI 95%)**

*% per PSU*			
Children	1.31 (0.86; 1.76)*	-0.25 (-0.70; 0.20)	-0.18 (-0.56; 0.20)
Adolescent	1.15 (0.68; 1.62)*	0.18 (-0.29; 0.65)	-0.19 (-0.59; 0.21)
Elderly persons	1.59 (0.93; 2.25)*	-0.32 (-0.98; 0.34)	0.29 (-0.27; 0.85)
*Mean income per PSU*	-0.04 (-0.18; 0.10)	0.08 (-0.06; 0.22)	0.66 (0.54; 0.78)*
*Education of the head of the family (%)*			
Illiterate	-0.64 (-1.83; 0.53)	0.47 (-0.70; 1.64)	0.59 (-0.41; 1.58)
Elementary	0.57 (0.05; 1.10)*	-0.54 (-1.06;-0.02)*	0.59 (0.14; 1.04)*
High school	1.19 (0.62; 1.75)*	-2.21 (-2.77;-1.65)*	1.09 (0.61; 1.60)*
Undergraduate and graduate	0.51 (-0.49;1.52)	-2.00 (-3.0;-1.00)*	1.31 (0.46; 2.15)*
*R*^*2*^	0.14	0.16	0.35

	**Northeast**		

	**Rice and beans** β (CI 95%)	**High energy** β (CI 95%)	**High Protein** β (CI 95%)

*% per PSU*			
Children	0.42 (0.15; 0.70)*	-0.34 (-0.60;-0.08)*	0.40 (0.15;0.66)*
Adolescent	0.68 (0.42; 0.95)*	-0.23 (-0.49;0.02)	0.42 (0.17;0.67)*
Elderly persons	0.47 (0.13; 0.81)*	0.09 (-0.24;0.41)	0.81 (0.49;1.12)*
*Mean income per PSU*	-0.05 (-0.15; 0.04)	0.49 (0.40;0.58)*	0.60 (0.51;0.69)*
*Education of the head of the family (%)*			
Illiterate	0.32 (-0.19;0.83)	-0.46 (-0.95;0.03)	1.00 (0.53;1.48)*
Elementary	-0.59 (-0.86;-0.31)*	0.55 (0.29;0.81)*	0.10 (-0.14;0.36)
High school	-1.74 (-2.08;-1.40)*	0.55 (0.22;0.87)*	0.22 (-0.10;0.53)
Undergraduate and graduate	-1.85 (-2.47;-1.22)*	0.53 (-0.07;1.13)	-0.69 (-1.27;0.11)

*R*^*2*^	0.21	0.24	0.19

	**Southeast**		

	**Rice and beans** β (IC 95%)	**Mixed** β (IC 95%)	

*% per PSU*			
Children	0.26 (-1.17; 0.71)	0.38 (0.02;0.74)*	
Adolescent	1.08 (0.65; 1.51)*	0.29 (-0.06;0.64)	
Elderly persons	0.74 (0.21; 1.26)*	0.97 (0.54;1.40)*	
*Mean income per PSU*	-0.05 (-0.14; 0.15)	0.75 (0.63;0.87)*	
*Education of the head of the family (%)*			
Illiterate	0.98 (-0.44; 2.40)	0.19 (-0.95;1.34)	
Elementary	-0.16 (-0.70; 0.37)	0.68 (0.25;1.11)*	
High school	-1.83 (-2.43; -1.24)*	1.20 (0.71;1.68)*	
Undergraduate and graduate	-0.88 (-1.67; -0.09)*	0.67 (0.02;1.31)*	
*R*^*2*^	0.16	0.39	

	**South**		

	**Rice and beans** β (IC 95%)	**Mixed** β (IC 95%)	

*% per PSU*			
Children	0.30 (-0.18;0.79)	0.41 (-0.02;0.84)	
Adolescent	0.56 (0.09;1.03)*	0.51 (0.09;0.93)*	
Elderly persons	0.54 (-0.06;1.14)	0.13 (-0.4;0.66)	
*Mean income per PSU*	-0.41 (-0.58;-0.23)*	0.88 (0.72;1.03)*	
*Education of the head of the family (%)*			
Illiterate	-0.18(-2.26;1.89)	-0.53 (-2.38;1.31)	
Elementary	0.52 (-0.1;1.15)	1.00 (0.44;1.56)*	
High school	-0.36 (-1.07;0.36)	0.92 (0.29;1.56)*	
Undergraduate and graduated	0.19 (-0.77;1.15)	0.52 (-0.33;1.38)	

*R*^*2*^	0.17	0.29	

	**Midwest**		

	**Rice and beans** β (IC 95%)	**Mixed** β (IC 95%)	

*% per PSU*			
Children	-0.02 (-0.45;0.40)	0.36 (0.007;0.71)*	
Adolescent	0.66 (0.25;1.07)*	0.19 (-0.14;0.52)	
Elderly persons	0.44 (-0.13;1.01)	0.53 (0.06;1.00)*	
*Mean income per PSU*	0.06 (-0.08;0.21)	0.76 (0.64;0.88)*	
*Education of the head of the family (%)*			
Illiterate	0.33 (-0.87;1.54)	0.16 (-0.83;1.14)	
Elementary	-0.06 (-0.60;0.48)	0.36 (-0.09;0.80)	
High school	-1.52 (-2.11;-0.92)*	0.69 (0.21;1.18)*	
Undergraduate and graduate	-1.29 (-2.15;-0.43)*	0.33 (-0.38;1.03)	

*R*^*2*^	0.11	0.34	

* p-value < 0.05

In the South, Southeast and Midwest beyond of rice and beans pattern, other designed as mixed pattern with positive loadings for the groups of vegetables and leafy vegetables (ex.: lettuces, pumpkin, chayote); potatoes; other tuberous roots (ex.: yam, sweet potatos, beets); fruits; coconuts; breads; cakes and cookies; fish; milk and dairy; butter and margarine; sweetened beverages (soft drinks; juices and others); and diet and/or light products was also extracted (Table 2).

A higher proportion of children, adolescents, and elderly people in the geographic sectors were associated with the regional pattern in the North and with the rice and beans pattern in the Northeast. In the other regions, only the presence of adolescents was associated with the rice and beans pattern (Table 3).

Analyses were also performed for the whole country urban and rural strata, where the observed dietary availability patterns observed were similar to the patterns in the macro-regions, one based on the rice and beans and another featured as a mixed pattern (Table 4).

**Table 4:** Loading values according to dietary patterns in the strata urban and rural of the Brazil

	Rural		Urban	
**Food Groups**	**Mixed**	**Rice and beans**	**Rice and beans**	**Mixed**

Rice		0.62	0.60	
Beans		0.56	0.66	
Vegetables and leafy vegetables	0.53			0.58
Potatoes	0.66			0.61
Others tuberous roots	0.48			0.51
Flours (wheat, manioc and corn meal)		0.27	0.56	
Fruits	0.51			0.68
Nuts and coconut		-0.06		0.11
Pastry	0.45		0.46	
Breads	0.50			0.44
Cakes & cookies	0.31			0.42
Meats	0.33		0.39	
Poultry and eggs	0.39		0.35	
Fish		-0.12	0.21	
Milk and dairy	0.32			0.33
Butter and Margarine	0.49			0.40
Sugar		0.61	0.72	
Sweetened beverages	0.59			0.49
Oils and fats		0.65	0.64	
Caffeinated beverages		0.46	0.53	0.13
Products diets/light	0.32		-0.03	0.37

Eigenvalues	2.97	2.19	3.15	2.77

Variance explained %	63.0	37.0	65.0	34.4

Cronbach Coefficient Alpha	0.76	0.75	0.64	0.68

Kaiser's Measure of Sampling Adequacy	0.79		0.85	

National Household Budget Survey 2002/2003.

## Discussion

In all brazilian macro-regions, with the exception of the North region, the most important dietary availability patterns included foods of typical brazilian diet, such as rice, beans, flours (wheat, manioc, and corn meal), sugar, butter and margarine, and oils and fats.

A survey conducted in Rio de Janeiro, Brazil, indicated that the traditional combination of rice and beans as major staple foods was protective against obesity [2]. Other studies conducted in Brazil also observed a predominant brazilian traditional pattern in areas such as São Paulo, and Porto Alegre [2,3,13,14].

The North region has the highest fish availability, obtained mainly through fishing [15]. The regional pattern included beyond fish nuts and coconuts. Only this pattern in the North region was associated with the composition of the families assessed by the proportion of children, adolescents, and elderly (Table 4).

Composition of the families with a higher proportion of adolescents in PSUs was also associated with the rice and beans pattern. It is possible that the intake of high-energy snacks and beverages among adolescents takes place mainly outside home, and the HBS does not get these consumptions.

Higher levels of education and income influence food acquisition [16], and our analysis showed that higher socioeconomic status had weaker association with the rice and beans pattern but also had stronger association with mixed pattern.

High energy pattern identified only in the Northeast region was associated with elementary and high school educational levels and higher income and was characterized by the availability of items with high energy density and poor nutritional value (Table 4). Our findings are in contrast with other studies where high energy density diets was the preferred choice of low income and low education populations in the Northeast region of Brazil [17]. Also, Lenz *et al. *[18] analyzed the dietary patterns of a sample of women in the South region of Brazil and detected an inverse relationship between income and the consumption of rice, beans, sugar, roots, and tuberous roots.

A study conducted in ten countries in Europe also indicated that the composition of the household influences the dietary pattern [7] with a Mediterranean diet being associated with the presence of elderly people in the household.

The household purchase data can be a valuable tool for obtaining information on the food pattern of a population. HBS data could help to explain issues such as differences in dietary patterns, high-risk population groups on account of their nutritional habits and valuable tool for many purposes, including nutrition and agricultural planning and marketing strategies [5,19]. A limitation of HBS studies, particularly of those conducted in Brazil is the short time (only a seven-day purchase period). For that reason, only clusters of households could be analyzed. Additionally, HBS do not take into account the purchases of items eaten out-of-home. In this HBS, 35% of the adults had at least one item eaten out-of-home in the seven-day period under consideration [20] a percentage that is much lower than those observed in developed countries such as U.S.. Studies of food eaten outside home have showed that these food have more fat, salt and are less healthy than those eaten home [21,22]. Thus, an incomplete record of beverages, snacks, and fast-foods may have attenuated our associations related to traditional or regional dietary pattern.

The Brazilian traditional dietary pattern, usually considered to be healthy, is still important as a household food custom in all over the country. The relationship of this pattern with the presence of adolescents in the household and its inverse relationship with the higher levels of education and income emphasize the importance of the family composition and socioeconomic characteristics associated to dietary practices and custom.

Although data of HBS express the availability of foods rather than the consumption, the data are a useful tool to depict dietary options between large regions of country. Composition of the families, income and level of education of the head of the families has also influence on the purchased items. Therefore, data of the HBS could help to recognize different food choices, allowing a proper planning of food and nutrition policies in the countries with large heterogeneity along their regions.

## Competing interests

The authors declare that they have no competing interests.

## Authors' contributions

All authors contributed to the design of this study. NS contributed to analyze, interpret the data and to draft the manuscript. FSB conducted data analysis and interpretation. RS conducted data analysis and interpretation and drafting of the manuscript. RAP conducted data interpretation and drafting of the manuscript. All authors helped to realize ideas, interpret findings, and review the manuscript. All authors approved the final manuscript.
